# Multiplicity of Buc copies in Atlantic salmon contrasts with loss of the germ cell determinant in primates, rodents and axolotl

**DOI:** 10.1186/s12862-016-0809-7

**Published:** 2016-10-26

**Authors:** Adrijana Škugor, Helge Tveiten, Hanne Johnsen, Øivind Andersen

**Affiliations:** 1 Norwegian University of Life Sciences (NMBU), PO Box 5003, N-1430 Ås, Norway; 2 Nofima, PO Box 6122, N-9291, Tromsø, Norway; 3 Nofima, PO Box 5010, N-1430 Ås, Norway

**Keywords:** Bucky ball, Germ plasm, Oskar, Mexican axolotl, Germline, Balbiani body

## Abstract

**Background:**

The primordial germ cells (PGCs) giving rise to gametes are determined by two different mechanisms in vertebrates. While the germ cell fate in mammals and salamanders is induced by zygotic signals, maternally delivered germ cell determinants specify the PGCs in birds, frogs and teleost fish. Assembly of the germ plasm in the oocyte is organized by the single Buc in zebrafish, named Velo1 in *Xenopus*, and by Oskar in *Drosophila*. Secondary loss of *oskar* in several insect lineages coincides with changes in germline determination strategies, while the presence of *buc* in mammals suggests functions not associated with germline formation.

**Results:**

To clarify the evolutionary history of *buc* we searched for the gene in genomes available from various chordates. No *buc* sequence was found in lamprey and chordate invertebrates, while the gene was identified in a conserved syntenic region in elephant shark, spotted gar, teleosts, Comoran coelacanth and most tetrapods. Rodents have probably lost the *buc* gene, while a premature translation stop was found in primates and in Mexican axolotl lacking germ plasm. In contrast, several *buc* and *buc*-like (*bucL*) paralogs were identified in the teleosts examined, including zebrafish, and the tetraploid genome of Atlantic salmon harbors seven *buc* and *bucL* genes. Maternal salmon *buc1a*, *buc2a* and *buc2b* mRNAs were abundant in unfertilized eggs together with *dnd* and *vasa* mRNAs. Immunostained salmon Buc1a was restricted to cleavage furrows in 4-cell stage embryos similar to a fluorescent zebrafish Buc construct injected in salmon embryos. Salmon Buc1a and Buc2a localized together with DnD, Vasa and Dazl within the Balbiani body of early oocytes.

**Conclusions:**

Buc probably originated more than 400 million years ago and might have played an ancestral role in assembling germ plasm. Functional redundancy or subfunctionalization of salmon Buc paralogs in germline formation is suggested by the maternally inherited mRNAs of three salmon *buc* genes, the localized Buc1a in the cleavage furrows and the distribution of Buc1a and Buc2a in the Balbiani body during oogenesis. The extra-ovarian expression of salmon *buc* genes and the presence of a second zebrafish *bucL* gene suggest additional functions not related to germ cell specification.

**Electronic supplementary material:**

The online version of this article (doi:10.1186/s12862-016-0809-7) contains supplementary material, which is available to authorized users.

## Background

Segregation of the germline and the specification of germ cells to become gametes are fundamental features in the animal kingdom, but the primordial germ cells (PGCs) are determined by two different mechanisms; zygotic induction (epigenesis) or cytoplasmic inheritance (preformation). PGCs are induced from pluripotent cells by extracellular signals in species with the inductive mechanism that involves BMP signals and the transcriptional repressor Blimp1 in mice and Mexican axolotl (*Ambystoma mexicanum*) [1–4]. The inductive mode of germline determination is found in basal chordates, salamanders, turtles and mammals, and is thought to be the ancestral mechanism [5–7]. Germ cells within teleost fish, frogs, snakes and birds acquire their fate early in embryogenesis through maternally deposited germ cell determinants known as germ plasm [6, 8]. The germ cell-specific mRNAs and proteins include Dazl, Dead end (Dnd) and Vasa, which are crucial for germline formation [9–15]. The conserved genes are expressed in the PGCs of all vertebrates, but the mRNAs and proteins are found dispersed in mouse, turtle and salamander, while they are localized in the germ plasm within the Balbiani body of zebrafish, Atlantic salmon, *Xenopus* and chicken [5, 16–21]. This mitochondria-rich organelle is one of the first morphological markers of oocyte polarity, but shows considerable variability in composition, morphology, developmental timing and persistence among metazoans [22].

Germ plasm assembly and cell-autonomous PGC specification have been demonstrated to be controlled by a single gene in the model species *Drosophila*, zebrafish and *Xenopus* [23–27]. In *Drosophila*, *oskar* is necessary and sufficient for the assembly of germ plasm in the oocyte posterior pole and the early specification of PGCs named pole cells [28, 29]. Knock-down of *oskar* in the wasp *Nasonia vitripennis* resulted in disrupted germ plasm and no pole cells, but defects in somatic patterning suggest complex roles of Oskar outside of the germline as well [30]. Intriguingly, *oskar* was found to have predated the evolution of germ plasm in insects by the identification of an ortholog in the cricket *Gryllus bimaculatus* that is not required for germ cell formation or axial patterning, but is necessary for neural development [31]. Although no detectable homology, the function of Oskar is remarkable similar to zebrafish Buc and *Xenopus* Velo1, which are characterized by a conserved N-terminal BUVE (Buc-Velo) motif. The novel region interacts with the RNA-binding protein Hermes to initiate Balbiani body assembly probably by recruiting *buc* and other RNAs, including *dazl*, to the germ plasm [27, 32]. Zebrafish *buc* mutant oocytes failed to localize *dazl* mRNAs in the Balbiani body that resulted in embryos without animal-vegetal polarity [23, 26]. Furthermore, the *buc* mutants possessed excess micropyles causing polyspermy, whereas overexpression of zebrafish *buc* seemed to generate ectopic PGCs in the embryo [23]. In *Xenopus*, two splice variants of Velo1 probably play largely redundant roles and are essential for germ plasm formation and maintenance [24, 27].

Orthologs of *buc* have been identified in several teleosts, chicken and mammals, but our knowledge about its key role in germ plasm assembly and oocyte asymmetry is solely based on studies in zebrafish and *Xenopus* [23, 25–27]. Paralog loss has apparently occurred in the two model species after the whole genome duplication events in teleosts and in *X. laevis* about 350 and 40 Mya, respectively. Tandem duplicates of *buc* have been reported in mammals and pufferfish [23] suggesting additional functions of Buc besides germline specification. In this study we examined the evolutionary history of Buc and the fate of the gene in vertebrates lacking germ plasm. Whereas a functional Buc has been lost in primates, rodents and axolotl, we found duplicated genes in all teleosts examined and possible redundancy of the Buc paralogs was investigated in the tetraploid Atlantic salmon.

## Results

### Origin and distribution of *buc* in vertebrates

**Fig. 1:**
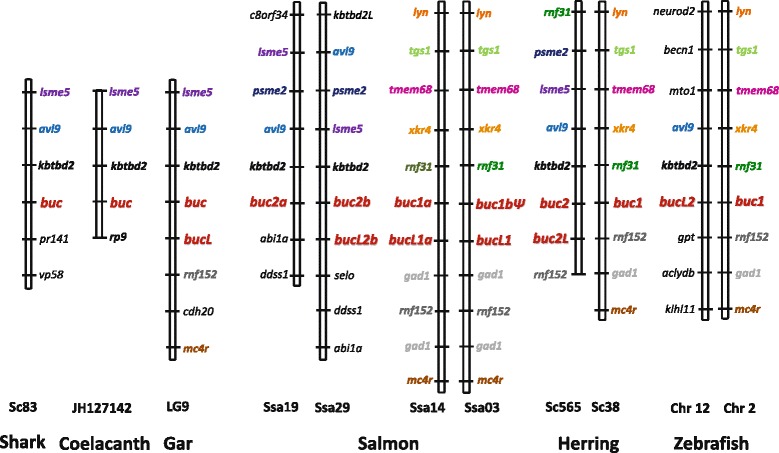
Conserved synteni of *buc* and neighbor genes in various fish species. Spotted gar *buc* and *bucL*-(like) genes have been duplicated in teleost fish, but several paralogs are lacking. Conserved syntenic genes are shown in bold, and orthologs are given in same color

**Fig. 2:**
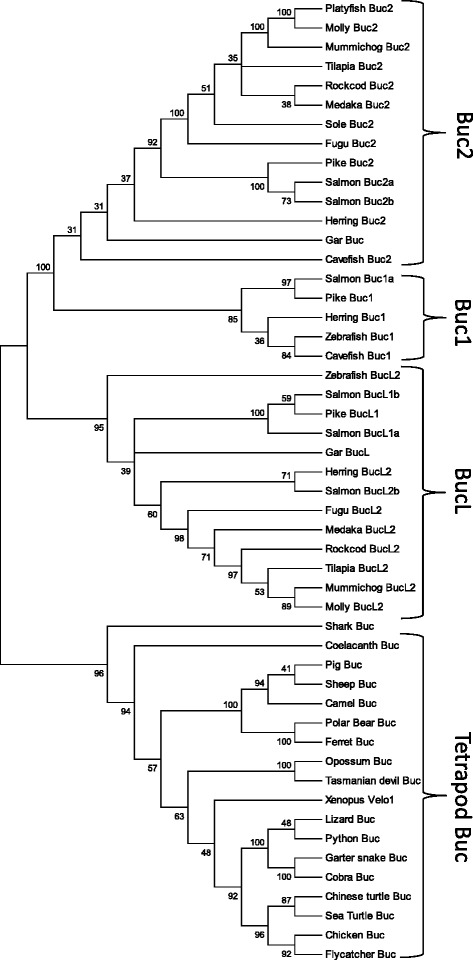
Unrooted phylogenetic tree of tetrapod Buc proteins and teleost Buc and BucL (−like) paralogs. The tree was generated using the ML method based on the JTT model. To evaluate the topological stability 100 bootstrap resamplings were made. All branches with less than 30 bootstrap confidence values were collapsed. Accession numbers are given in Additional file 3: Table S1

We searched for the *buc* gene in multiple chordate species representing key phylogenetic lineages by performing genomic BLAST search. No *buc*–related sequence was found in tunicates (*Ciona intestinalis* and *C. savignyi*), amphioxus (*Branchiostoma lanceolatum* and *B. floridae*), acorn worm (*Saccoglossus kowalevskii*) and in sea lamprey (*Petromyzon marinus*), while the elephant shark (*Callorhinchus milii*) genome was shown to harbor a single *buc* gene positioned next to the *kbtbd2*, *avl9* and *lsme5* genes (Fig. 1). The syntenic region was found to be conserved in Comoran coelacanth (*Latimeria chalumnae*), spotted gar (*Lepisosteus oculatus*), teleosts and in most tetrapods, but the number of *buc* genes varied. Cow and dog possess three and two genes copies, respectively, including pseudogenes (Additional file 1: Figure S1), while rodents seem to have lost the *buc* gene. The neighbor genes are conserved in mouse and rat, but are positioned next to a large rodent-specific gene cluster of vomeronasal receptors [33]. The single primate *buc* is a pseudogene with a premature translation stop after 20 amino acids (aa), while gorilla has an additional mutation in the translation start signal (Additional file 2: Figure S2). The complete *buc* gene was identified in the genome of Tasmanian devil (*Sarcophilus harrisii*), kangaroo rat (*Dipodomys ordii*), opossum (*Monodelhis domstica*), while a shorter Buc protein of 426 aa was predicted from platipus (*Ornithorhyncus anatinus*) (Additional file 3: Table S1). Blasting the axolotl genome against *Xenopus velo1* revealed a single base deletion that introduces a premature translation stop (Additional file 4: Figure S3). The truncated axolotl Buc of 190 aa shares only 52 % identity with *Xenopus* Velo1 and contains no N-terminal BUVE motif. The phylogenetic analysis of vertebrate Buc proteins showed that tetrapods form a separate clade together with the coelacanth, while the elephant shark branches off basal to the tetrapods (Fig. 2).

Spotted gar was shown to possess tandem repeated genes designated *buc* and *buc*-like (*bucL*) (Fig. 1), and additional duplications have likely occurred in teleosts during the third round of whole-genome duplication. Despite widespread paralog loss, a paralogon containing duplicated copies of *buc* and *bucL* was found in several species, including salmon, herring and zebrafish (Fig. 1). We named the *buc* paralog flanked by *rnf31* and *rnf152* as *buc1*, while *buc2* designates the paralog next to the *kbtbd2-avl9-lsme5* block. Consistently, the phylogenetic analysis separated Buc1 and Buc2 in two clusters, but the latter showed low bootstrap values (Fig. 2). Both paralogs were identified in salmon, herring, pike and cavefish, while *buc1* has been lost in most species examined (Fig. 2). Conversely, *buc2* is lacking in zebrafish, which has retained *bucL2* sharing only 30 % aa sequence identity with *buc1* (named *buc* in previous studies). The tetraploid Atlantic salmon genome was shown to harbor a total of seven *buc* genes positioned on the homeologous chromosomes Ssa14 - Ssa03 and Ssa19 - Ssa29 that comprised four *buc* genes, including the *buc1b* pseudogene, and three *bucL* genes (Fig. 1). All predicted salmon Buc proteins possess a conserved N-terminal BUVE-motif, but repeats were found in the BucL2b motif (Additional file 5: Figure S4).

### Maternal mRNAs of three *buc*, *dnd* and *vasa* in salmon embryo

**Fig. 3:**
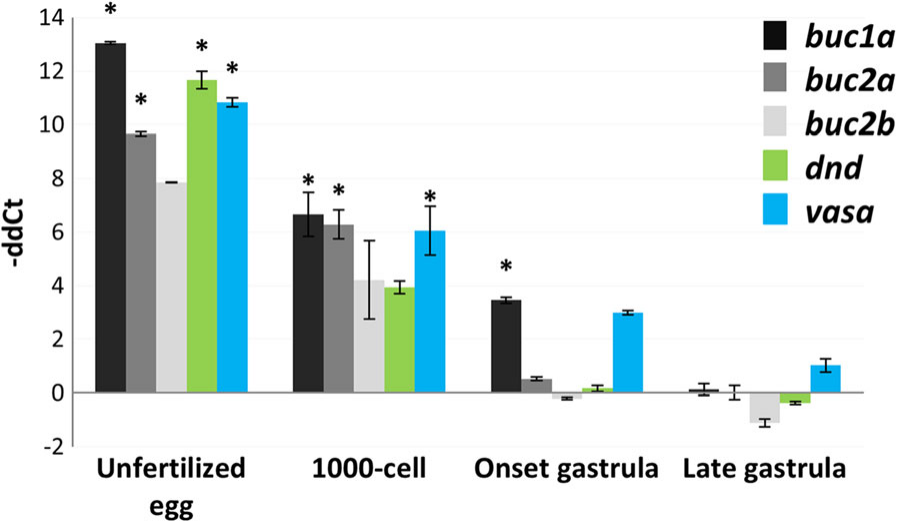
mRNA levels of salmon *buc1a*, *buc2a* and *buc2b* together with *dnd* and *vasa* during embryogenesis. The transcriptional levels of target genes were normalized to eEf1-a and presented as -ΔΔCt ± SE (*n* = 3). All stages were compared to the segmentation stage that follows late gastrulation. Significant difference is marked with * (*p* < 0.05)

We quantified the mRNA levels of salmon *buc1a*, *buc2a* and *buc2b* during embryogenesis using RT-qPCR. Maternal deposition of the three transcripts was evidenced by the high mRNA levels in unfertilized eggs that gradually decreased during embryogenesis (Fig. 3). Similar mRNA patterns were found in the germ plasm factors *dnd* and *vasa*, and only *buc1a* and *vasa* mRNAs were maintained at the onset of gastrulation.

### Salmon Buc1a and injected zebrafish Buc-GFP in cleavage furrows

**Fig. 4:**
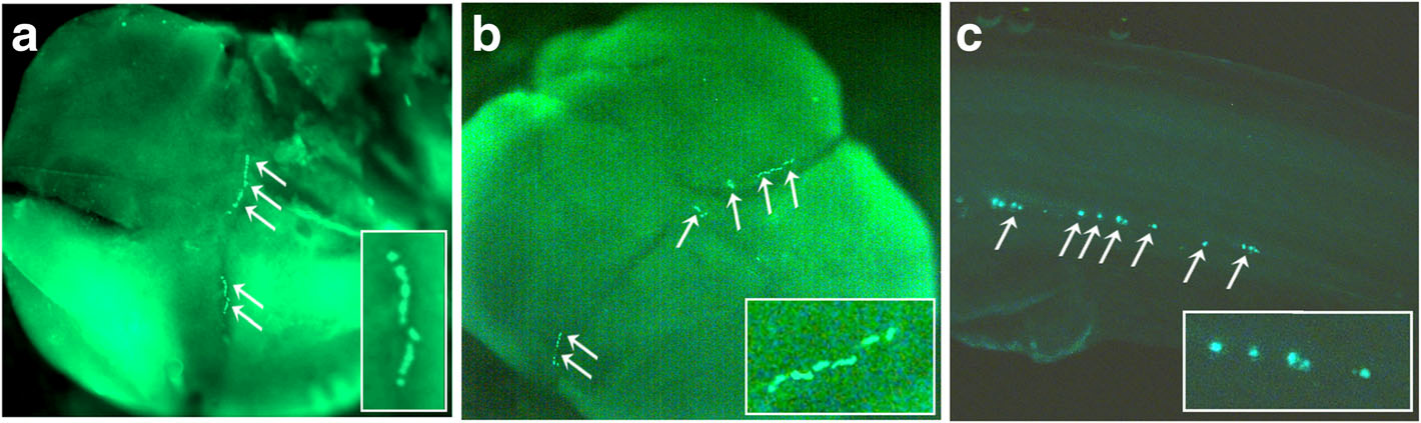
Salmon embryo at 4-cell stage with fluorescent signals in cleavage furrows from: **a** Immunostained endogenous Buc1a protein, **b** Zebrafish Buc-GFP injected in 1-cell salmon embryos. Magnified signals are shown in the white box. **c** Zebrafish Buc-GFP localized to putative PGCs in salmon embryos during segmentation. White arrows indicate fluorescent signals. Immunostained salmon embryo with secondary antibody only is shown in Additional file 8: Figure S5

Specific antibodies were produced against salmon Buc1a, Buc2a and Buc2b to localize the proteins in the developing embryo and in the ovary. Immunostaining of 4-cell embryos revealed Buc1a aggregates within the cleavage furrows similar to the localization of zebrafish Buc-GFP after injection of the mRNA construct in 1-cell salmon embryo (Fig. 4a, b). The intensity of fluorescence in the putative PGCs at segmentation stage (Fig. 4c) varied between embryos when different doses of the construct were injected. The position and morphology of the *buc*-expressing cells resembled the salmon PGCs labelled at the same stage using cod Nanos3-GFP mRNA reporter [34]. The Buc2a protein was not detected in the salmon embryo, while the Buc2b antibodies did not work satisfactorily.

### Gene expression of salmon *buc*, *dnd* and *vasa* during oogenesis

**Fig. 5:**
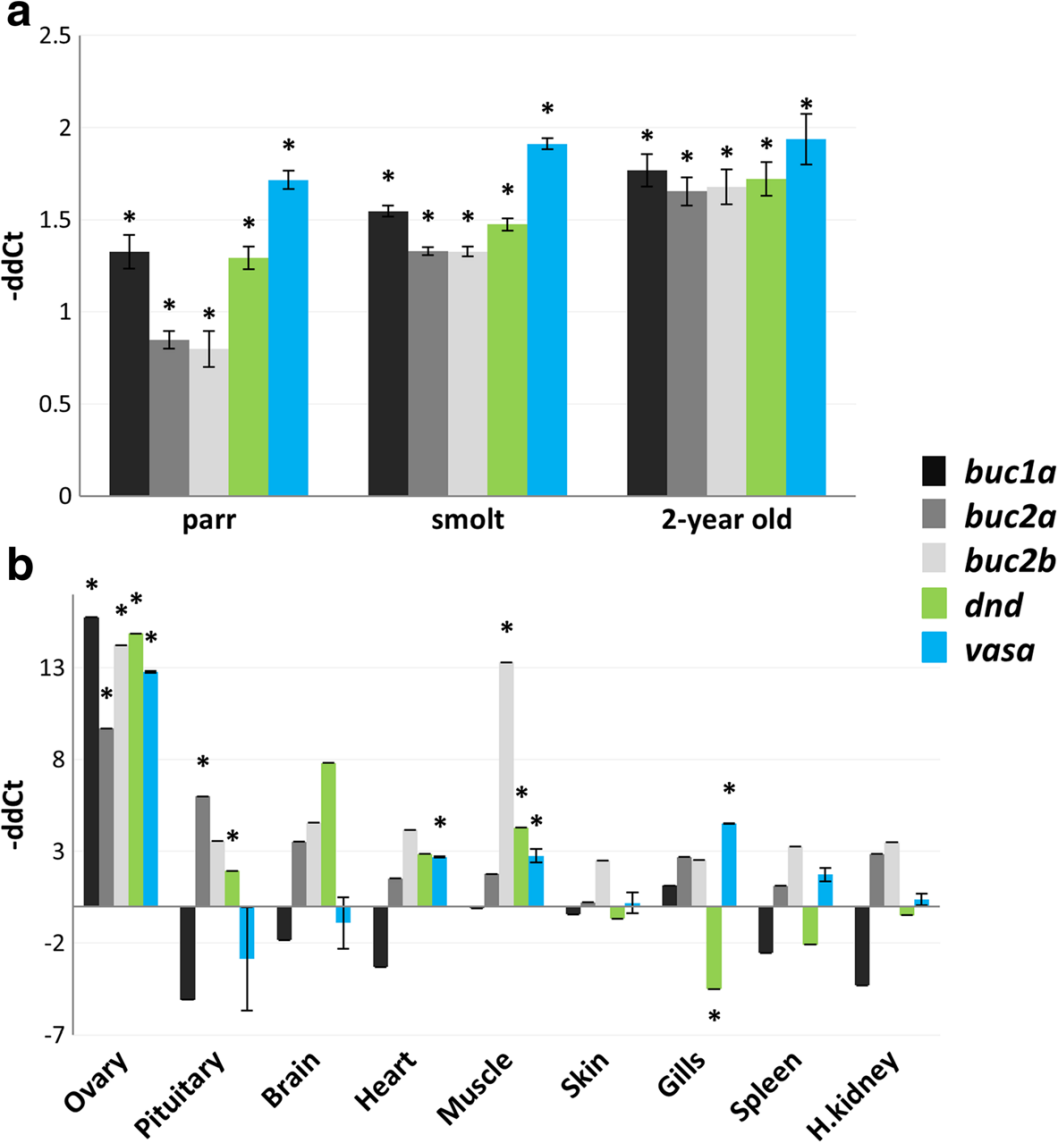
Gene expression of salmon *buc1*, *buc2a, buc2b, dnd* and *vasa*. **a** Ovarian mRNA levels during oogenesis at the freshwater (parr) and early seawater (smolt) stages compared to those in 3-year old ovary (*n* = 6). **b** Tissue expression in 2-year old females (*n* = 2). All tissues were compared to liver. The transcriptional levels of target genes were normalized to *eEf1-a*. Data are presented as –ΔΔCt ± SE. Significant difference is marked with * (*p* < 0.05)

Salmon *buc1a*, *buc2a* and *buc2b* were all expressed in the ovary during oogenesis with the highest mRNA levels measured in the maturing ovary of 2-year old females (Fig. 5a). The expression profiles of *dnd* and *vasa* were similar to the *buc* genes, including the higher levels in the maturing 2-year old ovary than in the juveniles and in the mature ovary of 3-year old females.

Extra-ovarian expression of the three salmon *buc* genes was measured together with *dnd and vasa* in 2-year old females (Fig. 5b). The three genes showed abundant expression in ovary, but the transcripts were widely distributed in other tissues. Low levels of *buc1a* mRNA were found in gills, while *buc2a* and *buc2b* displayed substantial expression in the pituitary and skeletal muscle, respectively. The *dnd* and *vasa* genes were highly expressed in the ovary, while tissues like brain and gills showed low mRNA levels.

### Localization of salmon Buc, Dnd and Vasa proteins during oogenesis

**Fig. 6:**
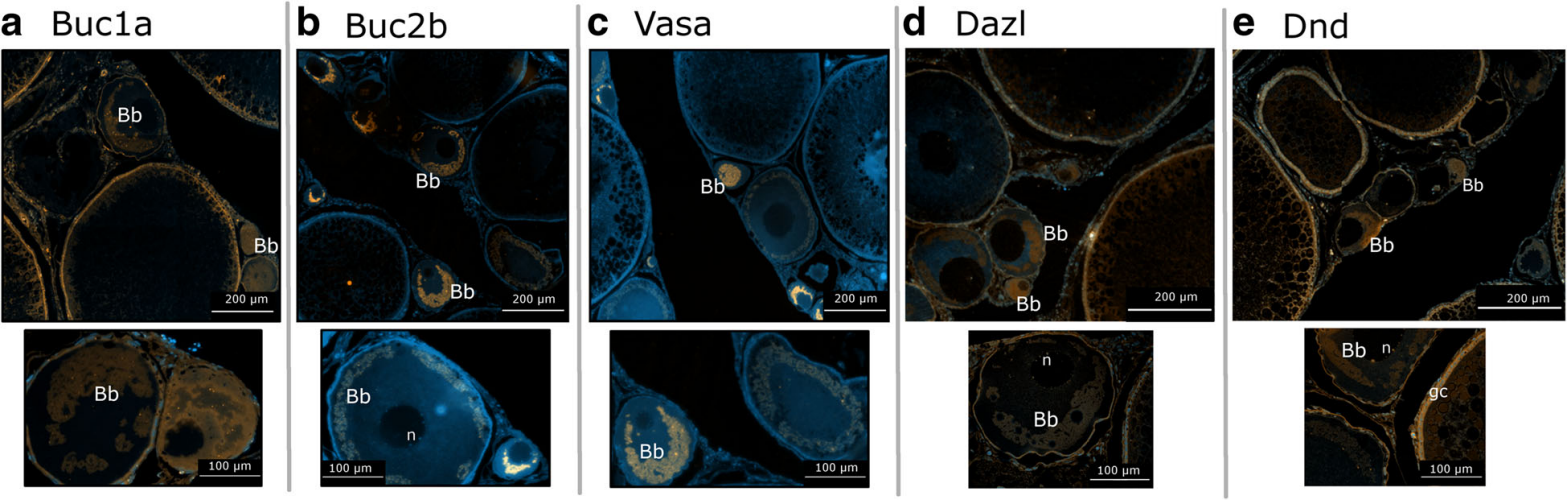
Immunohistochemical staining of ovarian sections from maturing 2-year old salmon. **a** Buc1a restricted to granular ooplasm of immature oocytes with magnified section below showing immature oocytes positively stained for Buc1a (orange). **b** Buc2a in ooplasm of immature oocytes with magnified view of Buc2b positive oocytes (orange) below. **c** Vasa positive signals (orange) in immature oocytes with magnified view below. **d** Dazl signals (orange) in Bb and nucleoli of immature oocytes and granulosa cells of more mature follicles with magnified oocyte positively stained for Dazl. **e** Population of oocytes at different developmental stages showing Dnd signals (orange) in both immature and cortical alveolus stage oocytes. Magnified view of Dnd signals is shown below. Proteins are stained orange and nuclei are stained blue with DAPI. Ovaries used to prepare the sections were collected from 3 different females. Bb- Balbiani body, gc- granulosa cell, n- nucleolus. Negative (secondary antibody alone) and positive (collagen staining) controls are shown in Additional file 8: Figure S5

**Fig. 7:**
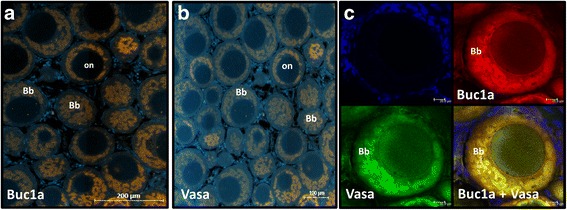
Immunohistochemical staining of juvenile ovarian sections. Adjacent sections were used for staining of (**a**) Buc1a (orange) and (**b**) Vasa (orange). **c** Co-localization of Buc1a and Vasa in the granular ooplasm of primary growth oocytes. Ovaries used to prepare the sections were collected from 3 different females. Nuclei were stained with DAPI (blue); Bb- Balbiani body, on- oocyte nucleus

Immunostaining of ovarian sections from 2–year old females showed both Buc1a and Buc2b signals at the periphery of the granular ooplasm of immature oocytes similar to the localization of Vasa (Fig. 6a, b, c). Dnd and Dazl were distributed in the granular ooplasm and nucleoli of immature oocytes, but were also visualized in the cytoplasma of granulosa cells surrounding the mature oocytes at the cortical alveolus stage (Fig. 6d, e). We further examined the location of Buc1a and Vasa in the juvenile ovary containing primary growth oocytes (stage 2a, 2b), and showed that the two proteins co-localized in the granular ooplasm within the putative Balbiani body (Fig. 7).

### TEM analyses of salmon ovary

**Fig. 8:**
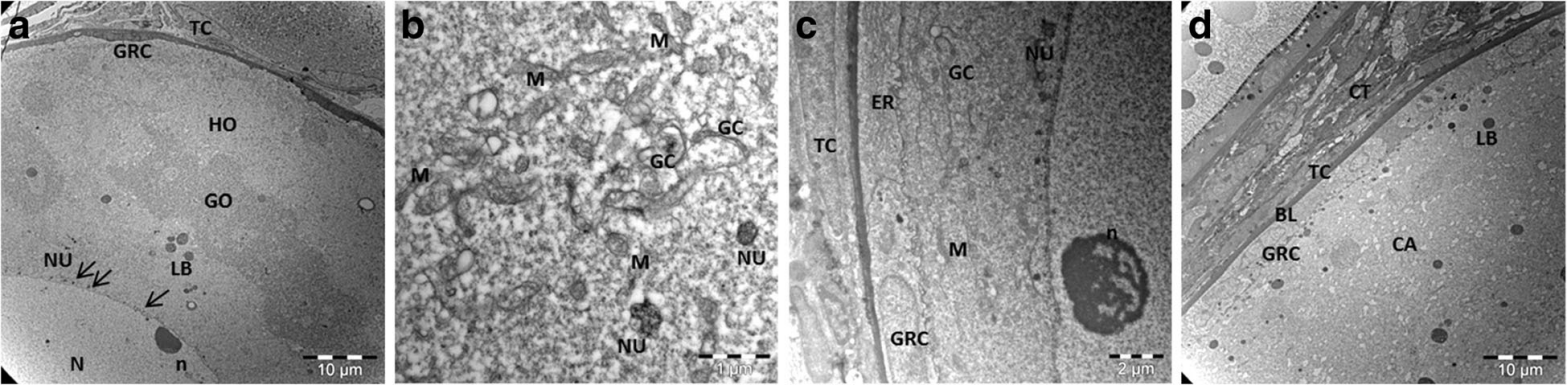
Ultrastructure of Atlantic salmon oocytes. **a** Stage 2 oocytes with granular ooplasm/Balbiani body. Black arrows indicate accumulations of nuage close to nuclear envelope and numerous nucleoli. **b** An area with aggregates of nuage and numerous mitochondria. **c** Ooplasm of stage 2 oocytes with endoplasmic reticulum and Golgi complex near the nuclear membrane. **d** Stage 4 oocyte surrounded with follicular cells. BL-basal membrane; CA-cortical alveoli; CT-connective tissue; ER-endoplasmic reticulum; GC-Golgi complex; GO-granular ooplasm; GRC-granulosa cell; HO- homogeneous ooplasm; LB-lipid body; M-mitochondria; NU-nuage; N-nucleus; n-nucleolus; TC-theca cell

To get a more detailed overview of oocyte structures in salmon ovaries at different developmental stages, we performed TEM analyses of ovaries from juvenile and 2-year old females (Fig. 8). Juvenile ovaries consisted exclusively of the primary growth stage 2 oocytes with irregular, electron dense nucleus and a cytoplasm without yolk granules (Fig. 8a). At this stage, accumulations of nuage were observed nearby nuclear envelope and numerous nucleoli, but also in the cytoplasm in close proximity to small vesicle and mitochondrial aggregates. TEM analysis revealed two distinct cytoplasmic zones in stage 2 oocytes; granular and homogenous (Fig. 8a). Interestingly, granular cytoplasm was rich in organelles comprising mitochondria, Golgi, ER and nuage (Fig. 8b, c). In contrast, ovaries from 2-year old females consisted of different oocyte populations. In addition to immature pre-vitellogenic oocytes, we observed a large population of cortical alveolus stage oocytes, which were substantially larger and contained yolk vesicles (cortical alveoli) of various sizes and showed more pronounced follicle epithelium (Fig. 8d). All oocytes were surrounded by follicle cells with characteristic, elongated nucleus (Fig. 8a, c, d). In rainbow trout, the Balbiani body disappear in the oocytes at stages 3 and 4 [35] corresponding to the lowered mRNA levels and the absence of salmon Buc proteins in the mature oocytes prior to spawning. Consistently, the zebrafish Buc protein was undetectable in stage III oocytes, while the mRNA levels were reduced at stage IV [23, 36].

## Discussion

This study traced the evolutionary origin of Buc back to more than 400 million years (mya) at the time cartilaginous fish appeared and lobe-finned fish (Sarcopterygii) separated from ray-finned fish (Actinopterygii). This agrees with the recent identification of ovarian *buc* in the Chinese sturgeon (*Acipenser sinensis*) representing the primitive Acipenseriformes with estimated origin time about 390 mya [37, 38]. The Balbiani body and specification pattern of the sturgeon PGCs were shown to closely resemble that of anurans, and injected zebrafish Buc-GFP accumulated at the cleavage furrows similar to our salmon study [39, 40]. Together with the localization of sturgeon *dazl* in the Balbiani body [41], the results suggest the involvement of Buc in the germ plasm assembly of this basal ray-finned fish. In cartilaginous fish, most researchers have identified germ cells first at late embryonic stages indicating zygotic induction [6, 42]. Furthermore, while the mode of germ cell specification in coelacanths is unknown, no mitochondrial cloud was found in oocytes of lungfish suggesting that germ plasm was absent in the common ancestor of tetrapods [7]. However, this should be confirmed by molecular analysis of the germ cell determinants available from recent studies of the coelacanth genome and lungfish transcriptome [43, 44]. It should be noted that cephalochordate PGCs were suggested to be epigenetically determined based on the first morphological identification of germ cells, whereas more recent studies have revealed maternal transcripts of *tudor7*, *vasa* and *nanos* co-localized in germ plasm that implies a function in germ cell formation [6, 45, 46]. In ascidian, the germline is silenced and primary cell lineages are determined by maternal *postplasmic/PEM* mRNAs [47–50], which might include a novel germ cell determinant yet to be found.

The repeated emergence of predetermined germ cells in different lineages suggests that the molecular regulator underlying maternal germ plasm assembly in principle is relatively easily acquired [7, 51]. The maternally inherited and zygotic inductive modes of germline formation have been studied in a few model species, and both mechanisms seem to require a single, or only a few, specific gene(s) expressed at the appropriate developmental stage, subcellular localization and dosage to direct germ cell specification [52–55]. The two strategies are used in amphibians wherein frogs (anurans) employ germ plasm, while salamanders (urodeles) do not [5, 17, 56]. *Xenopus* has lost the pluripotency genes *nanog* and *oct4*, but exhibits multiple gene copies of *Nodal*-related and *mix* regulating mesoderm development [57]. In contrast, axolotl has single copies of these genes and the PGCs are eliminated by excess Nodal levels and overexpression of *mix*, suggesting strict regulation of these genes in species with zygotic induction [1, 57, 58]. Conversely, germ plasm assembly in *Xenopus* is controlled by the Buc homolog Velo1, while only a partial *buc* sequence seem to have been retained in axolotl after the anurans and urodeles diverged from a common ancestor about 250 million years ago [59, 60]. Secondary loss of a functional Buc was also shown in rodents lacking the gene and in primates possessing only a pseudogene, whereas the gene has been conserved in non-primates and in turtles, which are using the inductive strategy [61]. The conserved syntenic region of *buc* has escaped chromosome rearrangements over a long evolutionary period and suggests a strong selective pressure on the region. We therefore propose that Buc played an ancestral role in germline formation and functions as the common regulator assembling the conserved germ plasm components in vertebrates with preformation. Altogether this suggests that the preformation mode was the ancestral mechanism and that the inductive mode has evolved repeatedly in vertebrates.

Tandem duplicated *buc* genes were found in spotted gar resembling the local gene duplications previously reported in several non-primate mammals [23]. The *buc* and *bucL* copies have been further duplicated in teleosts, but paralogs have apparently been lost after the third whole genome duplication event. In zebrafish, the Buc1 paralog is necessary and sufficient for germ plasm assembly and oocyte polarity [23], while the BUVE motif is not conserved in BucL2 and is probably not involved in germline formation. Most teleosts seem to have lost Buc1, and the Buc2 paralog has probably acquired the role as germ cell determinant, while sub- or neo-functionalization of Buc1 and Buc2 might have occurred in species possessing both paralogs. Consistent with a function in germ plasm assembly, the salmon *buc1a* paralog was shown to be maternally inherited, and the Buc1a protein was localized to the embryonic cleavage furrows similar to that of injected zebrafish Buc-GFP, but also endogenous zebrafish Buc [36]. Further, salmon Buc1a co-localized with Vasa in early oocytes in agreement with zebrafish Buc1 and Vasa [36]. The involvement of several salmon paralogs in the germline formation is supported by the co-localization of salmon Buc1a and Buc2a in the putative Balbiani body during oogenesis and by the maternal mRNAs in the salmon embryo. Dosage balance has been described as an important process for the retention of duplicate genes after whole genome duplication events [62–64]. While overexpression of *buc1* in the zebrafish embryo resulted in ectopic germ cells [23], appropriate mRNA levels are probably maintained by the three *buc* genes in the salmon embryo. Contrasting with the restricted expression of zebrafish *buc1* in females, the extra-ovarian expression of the three salmon genes suggests additional functions not associated with germline formation.

## Conclusions

Whereas zygotic induction has been thought to be the ancestral mechanism of germ cell specification, the long evolutionary history of Buc and the presence of maternal germ plasm in several basal vertebrates suggest that Buc played an ancestral role in germline formation. We propose that Buc functions as the common regulator of germ plasm assembly in vertebrates and has either been lost or has acquired a somatic function in lineages without germ plasm. In teleosts, duplicated Buc proteins are probably involved in both germline formation and somatic functions. Possible subfunctionalization or redundancy of the salmon Buc proteins in germ cell specification warrants further studies by targeted gene knockout.

## Methods

### Identification of *buc* genes and conserved synteny

Buc sequences were retrieved by searching the databases http://www.ncbi.nlm.nih.gov, http://www.ensembl.org (release 84), http://www.ambystoma.org/research/salamander-genome-project (axolotl, *Ambystoma mexicanum*), http://www.uniprot.org/taxonomy/7739 (amphioxus, *Branchiostoma floridae*), http://mosas.sysu.edu.cn/genome/gbrowser_wel.php (*Branchiostoma belcheri*, http://www.uniprot.org/taxonomy/10224 (acorn worm, *Saccoglossus kowalevskii*). Accession numbers are given in Additional file 3: Table S1. Unannotated genes were identified by BLAST using known orthologs as query sequences and by examination of conserved flanking genes. Downloaded sequences were controlled by examination of sequence homology and presence of a conserved BUVE motif. Syntenic blocks were identified by searching for conserved neighbor genes in the given databases.

### Phylogenetic analyses

A phylogenetic tree of vertebrate Buc was constructed using the Maximum Likelihood (ML) method. A total of 50 Buc peptide sequences from different species were imported to Mega6 [65]. Multiple sequence alignments were made using MUSCLE, and positions containing gaps and missing data were eliminated (Additional file 6: Figure S6). The best model for the alignment was chosen using the ML option (“Find the best DNA/protein models”) provided by the software. The model with the lowest BIC (Bayesian Information Criterion) scores was chosen as it best describes the substitution pattern. Based on the BIC values the JTT (Jones-Taylor-Thornton) model was chosen [66]. To evaluate the tree topological stability, 100 bootstrap resamplings were made and all branches with less than 30 bootstrap confidence values were collapsed. The unrooted tree with the highest log likelihood was presented.

### Fish and tissue collection

All fish material, including unfertilized eggs and sperm, were provided by AquaGen salmon breeding company (Trondheim, Norway). Fertilization was performed at the Aquaculture research station (Tromsø, Norway) according to the general protocol for salmon, and the fertilized eggs were incubated at a temperature of 5–6 °C. The following stages were sampled for analyses: 1) unfertilized eggs and 2) 1000-cell stage 3) onset of gastrula 4) late gastrula and 5) segmentation stage. The embryonic stages were calculated by multiplying the incubation temperature (in °C) with the number of days since fertilization. Three biological replicates of eggs and embryos were submerged in RNAlater (Ambion, Austin, Texas, USA) to be used for gene expression analyses.

Six females from each developmental stages of freshwater parr (25 g), seawater smolt (80 g) and adults of 2- and 3- years were anaesthetized in metacain (MS-222, Sigma-Aldrich, Oslo, Norway) and sacrificed before ovaries were removed. Additional organs (pituitary, heart, brain, muscle, head kidney, skin, gills and spleen) were collected from two 2-year old females for examination of extra-ovarian gene expression. All tissues were carefully excised and stored in RNAlater (Ambion, Austin, Texas, USA) according to the manufacturer’s protocol. For immunohistochemistry, ovaries from three biological replicates of 80 g juvenile and 2-year old females were fixed in 4 % paraformaldehyde (PFA), dehydrated in series of ethanol and finally stored in 70 % ethanol at −20 °C.

### RNA extraction, cDNA synthesis and quantitative real-time RT-qPCR

Total RNA was extracted from salmon tissues, eggs and embryos using TRIzol (Life Sciences) and PureLinkTM RNA mini kit (Ambion). On-column DNase treatment was performed using PureLinkTM DNase (Life Sciences) to remove traces of DNA and impurities. RNA concentration and purity was measured by NanoDrop ND-1000 spectrophotometer (Thermo Fisher Scientific, Wilmington, USA).

cDNAs were made using the AffinityScript cDNA synthesis kit (Agilent Technologies) in a 20 μl reaction system according to the manufacturer’s instructions. For tissue samples, 200 ng total RNA was reverse-transcribed into cDNA, whereas 50 ng was used for eggs and embryos. Gene expression levels were quantified by real-time qPCR. The PCR primers (Additional file 7: Table S2) were designed using the Primer3 software and synthesized by Life Technologies. *Eukaryotic elongation factor 1-alpha* (*eEf1-a*) was used as a reference gene for calculating embryonic and ovarian mRNA levels. Melting curve analysis revealed that each primer pair produced only one product. Efficiency was checked from tenfold serial dilutions of cDNA for each primer pair. A 2 × SYBR® Green PCR Mastermix (Roche Diagnostics, Mannheim, Germany), 0.8 mM of each primer, and 4 μl of 1:10 diluted cDNA template were mixed in 12 μl reaction volumes. PCR was performed in duplicates in 96-well optical plates on Light Cycler 480 (Roche Diagnostics, Mannheim, Germany) under the following conditions: 95 °C for 5 min (preincubation), 95 °C for 5 s, 60 °C for 15 s, 72 °C for 15 s (amplification), followed by 95 °C for 5 s and 65 °C for 1 min (melting curve); 45 amplification cycles were performed. Relative expression of mRNA was calculated using the ΔΔCt method [67]. Differences between control and tissues at different developmental stages were assessed with Student’s *t*-test (*p* < 0.05).

### Immunohistochemistry (IHC)

Specific antibodies against the three salmon Buc proteins were produced in rabbits by injection of KLH (keyhole limpet hemocyanin)-conjugated synthetic peptides derived from protein-specific epitopes following standard protocol (GenScript, Hong Kong). Similarly, antibodies against salmon Dazl and Dnd were produced against N-terminal epitopes in rabbits by GL Biochem Ltd (Shanghai, China). IHC was carried out on LR-white plastic (London Resin Company, EMS, England) 2 μm thick ovarian sections. Permeabilization step was performed with 0.1 % Triton X-100 in phosphate buffered saline-PBS (Sigma-Aldrich, Oslo, Norway) for 20 min. Unspecific binding sites were blocked using 5 % skimmed milk diluted in PBST (PBS with 0.1 % Tween-20) for 3 h. The sections were incubated over night at 4 °C with the Buc1a, Buc2b, Vasa, Dnd and Dazl primary antibodies diluted in PBST and 2 % skimmed milk. Negative controls were incubated with secondary antibodies only, whereas positive control was incubated with salmon specific collagen type 1 (Biologo, Germany) (Additional file 8: Figure S5). After washing in PBST, the sections were incubated with DyLight 549 (Jackson Immuno Research) secondary antibody diluted 1:400 for 2 h. Final PBST wash was carried out before mounting in Dako fluorescent mounting medium (Glostrup, Denmark). All images were taken using a Zeiss Axio Observer Z1 microscope.

4-cell stage embryos used for IHC with Buc2b antibody were dechorionated and incubated in pre-chilled acetone for 20 min at −20 °C. This was followed by PBST wash and blocking in 5 % sheep serum for 2 h at room temperature. After blocking, embryos were incubated with the primary antibody as described above. Next, embryos were thoroughly washed in PBST before the secondary antibody Alexa Fluor 488 (Life Technologies) was applied. This was followed with PBST washes and imaging using Lumar.

### Microinjections of zebrafish *buc* mRNA

A zebrafish *buc-GFP* mRNA construct was kindly provided by Dr. Roland Dosch, University of Geneva, Switzerland) [35]. Salmon embryos at the 1-cell stage were injected with ≈ 200 ng *buc* mRNA in 0.05 % phenol red solution and incubated at 5–6 °C. A minimum of 100 embryos was injected at the 1-cell stage, and at least 60 % of them expressed Buc-GFP construct in the cleavage furrows. At segmentation, the number of embryos expressing Buc-GFP decreased to about 40 %. Images were taken at the 4-cell and segmentation stages using Lumar.

### Transmission electron microscopy (TEM)

Fragments of ovarian tissues were fixed in 25 % glutaraldehyde solution for 5 h at room temperature, and post-fixed in a 1 % osmium tetroxide in 0.1 M cacodylate buffer for 1.5 h at room temperature. Samples were then dehydrated in a graded acetone series, and infiltrated and embedded in LR White (London Resin Company, EMS, England). Ultrathin sections (70 nm) were obtained with a Leica EM UC6 Ultramicrotome. The sections were stained with 4 % uranyl acetate and 1 % potassium permanganate for 5 min, examined and photographed with a FEI Morgagni 268 transmission electron microscope operated at 80 kV.

## Additional files

Additional file 1: Figure S1. Chromosome localization (A) and sequence alignment (B) of *buc* and *buc*-like (*bucL*) genes in cow and dog. Putative pseudogenes (Ψ) contain premature stop codons (*), which are ignored in the protein prediction. (DOCX 70 kb)
Additional file 2: Figure S2. Partial *buc* genes from eight primates including with predicted human N-terminus (above) compared with ferret *buc* and predicted protein (below). The inserted A in primates results in a premature stop, while the deletion of the inserted base would introduce another stop as shown in italics on top. Translation start codon is mutated in gorilla. Chromosomal positions of the primate *buc* genes: Human; Chr5:129170048, Chimpaneze; Chr5:129813080, Gorilla; Chr5:112518743, Orangutan; Chr5:130553556, Baboon; Chr6:122901841, Gibbon; Chr1:28463206, Macaque; Chr6:125569040, Vervet; Chr23:32116145. (DOCX 14 kb)
Additional file 3: Table S1. Accession numbers of vertebrate Buc and BucL proteins. (DOCX 17 kb)
Additional file 4: Figure S3. Partial Mexican axolotl Buc predicted from two contigs identified in the genome by Blasting against *Xenopus* Velo1. Sequence alignment revealed a single base deletion introducing a premature translation stop. The partial axolotl Buc shares 52 % identity with *Xenopus* Velo1. (DOCX 15 kb)
Additional file 5: Figure S4. Conservation of the N-terminal end in A. Buc of various vertebrates, and B. BucL (−like) of zebrafish, salmon and spotted gar. Well conserved positions are shown in bold. (DOCX 15 kb)
Additional file 6: Figure S6. Multiple Buc sequence alignments from different species were obtained using MUSLE (multiple sequence comparison by log-expectation) (www.ebi.ac.uk/Tools/msa/muscle/). The alignments were imported to GenDoc for visual inspections. (PNG 891 kb)
Additional file 7: Table S2. Primer list for real-time qPCR (F-forward, R-reverse). (DOCX 16 kb)
Additional file 8: Figure S5. A. Negative control (secondary antibody alone) of immunostained salmon embryo at 4-cell stage shown in Fig. 4. B. Negative control (secondary antibody alone) and C. Positive control (collagen staining) of immunohistochemical staining of ovarian sections from maturing 2-year old salmon shown in Fig. 6. (DOCX 1930 kb)
