# Supplementary material for: Multiplicity of Buc copies in Atlantic salmon contrasts with loss of the germ cell determinant in primates, rodents and axolotl
Source: BMC Evol Biol. 2016 Oct 26;16:232. doi: 10.1186/s12862-016-0809-7 (PMC5080839; doi:10.1186/s12862-016-0809-7)
Supplement: Additional file 1: Figure S1. — Chromosome localization (A) and sequence alignment (B) of buc and buc-like (bucL) genes in cow and dog. Putative pseudogenes (Ψ) contain premature stop codons (*), which are ignored in the protein prediction. (DOCX 70 kb) [file 12862_2016_809_MOESM1_ESM.docx]

**Additional file 1: Figure S1**

**A.**


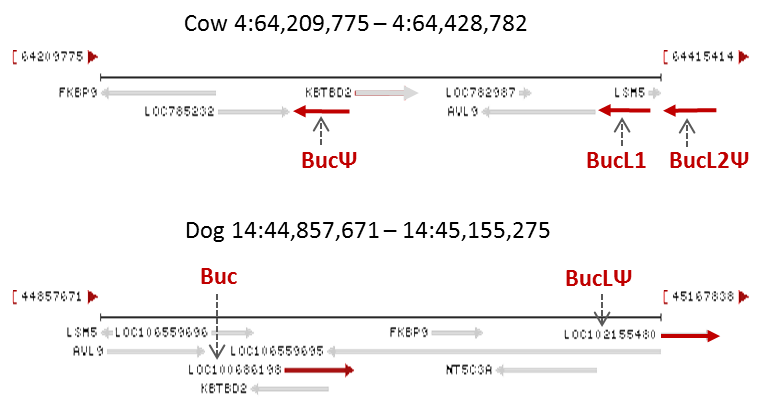


**B.**

Cow_BucΨ INWP-ENNEKCESNNSRSFFYVPPKAQQPRLGPWYQNPACNPLSISGAGGFRNGSLYFPC

Cow_BucL1 MNSS-GNTENFEGSNSRSFFYVHPMAQQPYLGPWYQNPVCNPLSIPGA-GFTNGSLYFP-

Cow_BucL2Ψ --------EAYRGSTSRPFFYVPVMAQKPHLGPWYQNPAYNPLSIPGAGGFTSGSLYFP-

Dog_Buc MNSSSENSAHGEGGTARPFFYVHAVGPPPYPSPWYQNLPANPCCVPGA-GFRNGSLYFPY

Dog_BucLΨ MNSSSENSAHGEGGTARPFFYVHAVGPPPYPSPWYQNLPANPCCVPGA-GFRNGSLYFSY

Cow_BucΨ SMVLSEYPAFLVPQSSLPTILN*****RPVVPISYNVAQFQRYGGYGEEMKMKHTHTE--PQQA

Cow_BucL1 -VVLSEYPAFLVPQSPLPTTVNRRSIVPMFYNTAQFRQYGGYWEKMKTKDTQTEAEPQQA

Cow_BucL2Ψ -VVLNEHPTFLIPQSLLPTTLDQRSMVPVFYNMAQFQQYGGCWKEMKTKDTQTEAEPQQA

Dog_Buc SVVLSEYPGFLIPQSPLPTAFYRR---PMFCNTAQFRQCSGYGQKTNTKETQTE--PHQA

Dog_BucLΨ SVVLREYHGFLIPQSPLPTALTKR---PVLCNTAQFCQCSGYGKKTNTKETQTE--PHQA

Cow_BucΨ ENMNKNKMCTQKAKPMT*****EG*****LVSLILTLKLTTFIKK-----HMGL*****FSLSRRGS*****MARA

Cow_BucL1 ENLNEK-------QDMHSEG----------------------------------------

Cow_BucL2Ψ ENLDKK-------QDVNSEG----------------------------------------

Dog_Buc ENMTQK-------QDTHSEGDRVTSILTSNIDTKAGK-PEATGRVLSSVVQ-KEPHPESP

Dog_BucLΨ ENMTQK-------QDTHSEGDRVTSILTSNIDRKTGKTPKATDRVLSSVVQKKEPHPESP

Cow_BucΨ LLITLYRNAPQGSYASKKELRRLEQGKGSSMIKFWKSLKQTVGLYDVAYGTTMPENVLQH

Cow_BucL1 -------DSP-------------EVGKVTSIPA--STSNEMMPSYDVVYGKDMPQKEVPQ

Cow_BucL2Ψ ------------------------------------------------------------

Dog_Buc SPNIVYRISPQGHYVPEKAKTRPEQSKGCPVTQFWKTLKETIRLYNLTYGKTMPENLVQH

Dog_BucLΨ SHNIVYRISPQEHYVPEKAKMRPEQSKGCPIIQFWKTLKETIHLYNLTYVKALPENLV*****H

Cow_BucΨ NGISQSSCKSRGVLYNLMRVETTL-----------------------------HIKMNTE

Cow_BucL1 NGISQSFCESGGMLYNSYEGRDRM-----------------------------NLDEKKK

Cow_BucL2Ψ ------------------------------------------------------------

Dog_Buc SGISQSPCESRSVLYNRHEGADSITGKDDESTIWSKQCHDVKHDEVVKSGSIRDMNLGKE

Dog_BucLΨ SGISQSPCESRSELCNRHEGADSITCKDEESTIWSEQCHDVKHDEVVKSGSIRDMNLGKE

Cow_BucΨ LFSQSSILML*****DMMRC*****SKNLS*****R*****TSMRKIRDMQLFPKPTTSE*****QR*****DPEYPEFKTVLV

Cow_BucL1 RYSALS----------QNPPPPNGRDDIQNTRNSKLYQ--SAGDMNKLHQERA------L

Cow_BucL2Ψ ------------------------------------------------------------

Dog_Buc KGCAAVPQFL------SSPGEAKDRDEIQDTLNSSLCQ--SSRDGNQLQQERP------F

Dog_BucLΨ KGCAAVFQFL------SSPGEAKDRDEIQDTLNSSLCQ--SSGDGNQLQQERP------F

Cow_BucΨ CSSMEAVKDLSLHSESSQKPFTAGESMPKNSSGSHGSLETV--GGEEESNSYPEMPVPSP

Cow_BucL1 CTSMEAVKDLSLCSESSQKPFTAGESMPENSSRSHGSPETV--GEEVESNSYPVMAIPSP

Cow_BucL2Ψ -----NSHDMRVVTS-----------------------------------------IPSP

Dog_Buc CSSNKAIEDLSLQSK-SQSPISFGENMPDNGSGGHGLLVKVDEGEVVEMHSCPQSYVPPP

Dog_BucLΨ CSSNKAVEDLSLQSR-SQSPISFGESMPDDGSGDHGFPVKVDEGEVVEMHSCPQSYVPPP

Cow_BucΨ PCLAQVSKEDEGIQCNMSW*****IEI*****SGNSPESSPGSSRKGAEQMSVGSRNQDEVYEVENNG

Cow_BucL1 PSLAQVSKEDEGIQCDWTWWTEVQPGNSPESSPGSSRKGAEQMSVGSQNQDEVDEVENNG

Cow_BucL2Ψ AWLTQSNKVGEGIQCDMRYAAKQT----------ECRNQDKQGYEGEEE--------NSN

Dog_Buc TSLAQFSQVSEGIQCDMSQWQDAEHEQSPESSP-ESRKTSEEGAVRCGELDEV--VVRDG

Dog_BucLΨ TSLAQFSQVSEGIQCDTSQWQDAEHEQSPEPSP-ESRKTSEEGAVRCGELDEV--VVRDG

Cow_BucΨ CEGEVPSPTWLAQVNK-VDEGIQYNMSCF-EAQVEKSPSKCPPRGEETVSDSKTRGSWKQ

Cow_BucL1 CEGEVPSPTWLAQVNK-VDEGIQCDMGYS-EAQVEKSPQQMPPRGEETVSDSKTRGSWEQ

Cow_BucL2Ψ YEGQGSSPTCLAQVNK-VDEGIQCDMSCY-KAQVEKSPQQMPSRGEETASDSKTRGSWKK

Dog_Buc FPKYVPNPAWLAQMNKGVDAGIQCDGSWWQDAELEKSPDQMPSKDEESSPACKTEGSQGK

Dog_BucLΨ FPKYVPNPAWLAQMNKGVDAGIQCDDSWWQDAELEKSPDQMPSKDEESSPACKTEGSQRK

Cow_BucΨ PITNRKLSADKEEMIMNDETGEVYNENLKRCAKIKKTLKGRKLKELSSFSNVKTAYLLKK

Cow_BucL1 PITNRKLSADKEEMIMNDETGEVYNENLKTCAKIKKTVKGRKLKELSSFSNVKTAYLLKK

Cow_BucL2Ψ QLQTGN*****A---WIKMK*****S*****MMRLRRHIMRT*****KGVQKL---KRL*****KAGS*****KT*TASQMLRQ

Dog_Buc TIRTGELNTD-EEMTRKDETEEEEHENFKKCADIQKSVTGRKQTSLSNFSSGNTGYLVRE

Dog_BucLΨ TIRTGELNTD-EEMTRKDETEEEEHENFKKCADIQKSVTGRKQTSLSNFSSGNTGYLVKK

Cow_BucΨ RVALTIVLPEDSEDSELEEE--GDMDEVGCLLEEVSPRGPLTSFKGRSYHEAGRIIRMPP

Cow_BucL1 SAVLTIVLPEDSEDSELEEE--GDMDEVGCLLEEVSPQSPVTSSKGRSYHEAGRIIRMPP

Cow_BucL2Ψ PIC*****RKLLS*****PLCFSRIQKTLSWKRRVTWMR*****DASL-------KK*****VHRAL*****HLPQEGLI

Dog_Buc NAASNTVLLEDSEDCDFEEEVEGEMEELDCLFAEVSPLGPVASSKGQIYHKAGRIIRMPP

Dog_BucLΨ NAASSTVLPEDSEDCDFEEEVEGEMEELDCLFAEVSPLGPVASSKGQIYHKAGRIISMPP

Cow_BucΨ ENSLSN---LW-----------YGPPEI

Cow_BucL1 ESSLPPQLMVWPTRNKCKLLQAYGECECVPAVYWKRQDGCESTGVRLRSRPTMANKEGLQ

Cow_BucL2Ψ TRLEGALSLPDL*****SGPPEISASFYKSVVNVSVSLLLRQDDCESTGVRRSKPTMAKSEGLQ

Dog_Buc ECCLPSQLLMWPTRNKNRLK--HGEYESIPVVC--KVTGQDG---RFRGECTTAMQEGLE

Dog_BucLΨ *****CCLPSQLLVWPTRNKNRLK--HGEYESIPVVC--KVTGQDG---RFRGECTTAMQEGLE

Cow_BucΨ

Cow_BucL1 SRRASHRPLECN

Cow_BucL2Ψ SRRASHKSLEC

Dog_Buc SKRASHKSWERNCQRWKTKVPYKVSNRFEADAVKFRK

Dog_BucLΨ SKRASHKSWER
