# Supplementary material for: Multiplicity of Buc copies in Atlantic salmon contrasts with loss of the germ cell determinant in primates, rodents and axolotl
Source: BMC Evol Biol. 2016 Oct 26;16:232. doi: 10.1186/s12862-016-0809-7 (PMC5080839; doi:10.1186/s12862-016-0809-7)
Supplement: Additional file 2: Figure S2. — Partial buc genes from eight primates including with predicted human N-terminus (above) compared with ferret buc and predicted protein (below). The inserted A in primates results in a premature stop, while the deletion of the inserted base would introduce another stop as shown in italics on top. Translation start codon is mutated in gorilla. Chromosomal positions of the primate buc genes: Human; Chr5:129170048, Chimpaneze; Chr5:129813080, Gorilla; Chr5:112518743, Orangutan; Chr5:130553556, Baboon; Chr6:122901841, Gibbon; Chr1:28463206, Macaque; Chr6:125569040, Vervet; Chr23:32116145. (DOCX 14 kb) [file 12862_2016_809_MOESM2_ESM.docx]

**Additional file 2: Figure S2**

*M N Y Y S K S S A Q F V G S N P R P Y stop I Q A M A H P F L G L W Y Q N P I Y*

M N Y Y S K S S A Q Y C R Q Q P Q T L L N T G Y G P S F P G S V V P E S H L stop

Human ATGAACTACTATTCAAAAAGCAGTGCACAAT**A**TTGTAGGCAGCAACCCCAGACCCTATT---AAATACAGGCTATGGCCCATC---CTTTCCTGGGTCTGTGGTACCAGAATCCCATCTATGA

Chimpaneze ATGAACTACTATTCAAAAAGCAGTGCACAAT**A**TTGTAGGCAGCAACCTCAGACCCTATT---AAATACAGGCTATGGCCCATC---CTTTCCTGGGTCTGTGGTACCAGAATCCCATCTATGA

Gorilla A**C**GAACTACTATTCAAAAAGCAGTGCACAAT**A**TTGTAGGCAGCAACCCCAGACCCTATT---AAATACAGGCTATGGCCCATC---CTTTCCTGGGTCTGTGGTACCAGAATCCCATCTATGA

Orangutan ATGAACTACTATTCAAAAAGCAGTGCACAAT**A**TTTTAGGCAGCAACCCCAGACCCTATT---AAATGCAGGCTATGGCCCATC---CTTTCCTGGGTCTGTGGTACCAGAATCCCATCGATGA

Baboon ATGAACTATTATTCAAAAAGCAATGCACAAG**A**TTGTAGGCAGCAACCCCAGACCCTATT---AAATGCAGGCTATGGCCCGGC---CTTTCCTGAGTCTGTGGTACCACAATTCCATCTATGA

Gibbon ATGAACTACTATTCAAAAAGCAGTGCACAAT**A**TTGTAGGCAGCAACCCCAGACCCTGTT---AAATGCAGGCTATGGCCCATC---TTTTTCTGGGTCTGTGGTACCAGAATCCCATCTATGA

Macaque ATGAACTATTATTCAAAAAGCAATGCACAAT**A**TTGTAGGCAGCAACCCCAGACCCTATT---AAATGCAGGCTATGGCCCAGC---ATTTCCTGGGTCTGTGGTACCACAATTCCATCTATGA

Vervet ATGAACTATTATTCGAAAAGCAATGCACAAT**A**TTGTAGGCAGCAACCCCGGACCCTATT---AAATGCAGGCTATGGCCCAGC---CTTTCCTGGGTCTGTGGTACCACAATTCCATCTATGA

Ferret ATGAACTCCTCTTCAGAAAACAATGCACATT-TTGAGGGCAGTAACGCCAGACCGTTTTTTTATGTGCATGCCGTGGGCCAGCAGACTTACCCGAGTCCTTGGTACCAGAATCCCAGCTATAAT

M N S S S E N N A H F E G S N A R P F F Y V H A V G Q Q T Y P S P W Y Q N P S Y N
