# Supplementary material for: Multiplicity of Buc copies in Atlantic salmon contrasts with loss of the germ cell determinant in primates, rodents and axolotl
Source: BMC Evol Biol. 2016 Oct 26;16:232. doi: 10.1186/s12862-016-0809-7 (PMC5080839; doi:10.1186/s12862-016-0809-7)
Supplement: Additional file 3: Table S1. — Accession numbers of vertebrate Buc and BucL proteins. (DOCX 17 kb) [file 12862_2016_809_MOESM3_ESM.docx]

**Additional file 3: Table S1.** Accession numbers of vertebrate Buc and BucL proteins

Atlantic salmon (*Salmo salar*): Buc1a, XP_013995965; BucL1a, XP_013995958, Buc1b, XR_001327509; BucL1b, XP_014046367, Buc2a, XM_014159853; Buc2b, XP_014036660; BucL2b, XP_014036659.

Northern pike (*Esox lucius*): Buc1, XP_010889676; BucL1, XP_010889677; Buc2, XP_010902006;

Cavefish (*Astyanax mexicanus*): Buc1, XP_007232162; Buc2, XP_007260081.

Zebrafish (*Danio rerio*): Buc1, NP_001243709 ; BucL2, XP_003199627.

Atlantic herring (*Clupea harengus*): Buc1, XP_012683306; Buc2, XP_012688635; BucL2, XP_012688636.

Platyfish (*Xiphophorus maculatus*): Buc2, XP_005810834.

Tilapia (*Haplochromis burtoni*): Buc2, XP_005941969; BucL2, XP_005941968.

Medaka (*Oryzias latipes):* Buc2, XP_004081403; BucL2, XP_011487418.

Amazon molly (*Poecilia formosa):* Buc2, XP_007565102, BucL2, XP_007565101.

Tongue sole (*Cynoglossus semilaevis*): Buc2, XP_008306554.

Fugu (*Takifugu rubripes*): Buc2, XP_011606429; BucL2, XP_011606431.

Rockcod (*Notothenia coriiceps*): Buc2, XP_010766020; BucL2, XP_010766019.

Mummichog (*Fundulus heteroclitus*): Buc2, XP_012738056; BucL2, XP_012738052.

Elephant shark (*Callorhinchus milii*): Buc, XP_007899090.

Spotted gar (*Lepisosteus oculatus*): Buc, XP_015210126, BucL, XP_015210136.

Comoran coelacanth (*Latimeria chalumnae*): Buc, XP_014346359.

Clawed frog (*Xenopus laevis*): Buc, NP_001082685.

Gartersnake (*Thamnophis sirtalis*): Buc, XP_013909729.

Pyton (*Python bivittatus*): Buc, XP_007431669.

Cobra (*Ophiophagus hannah*): Buc, ETE59467.

Green anole (*Anolis carolinensis*): Buc, XP_008111035.

Chinese soft-shelled turtle (*Pelodiscus sinensis*): Buc, XP_006124100.

Green sea turtle (*Chelonia mydas*): Buc, XP_007064417 .

Collared flycatcher (*Picedula albicollis*): Buc, XP_005041325.

Chicken (*Gallus gallus*): Buc, XP_015137153.

Opossum (*Monodelphis domestica*): Buc, XP_007489212.

Platipus (*Ornithorhynchus anatinus*): Buc, XP_007667979.

Tasmanian devil (*Sarcophilus harrisii*): Buc, ENSSHAP00000006719.

Polar bear (*Ursus maritinus*): Buc, XP_008684691.

Ferret (*Mustela putorius*): Buc, XP_004762675.

Camel (*Camelus bactrianus*): Buc, XP_010969389.

Sheep (*Ovis aries*): Buc, XP_011967555.

Pig (*Sus scrofa*): Buc, XP_003484094.
