# Supplementary material for: Multiplicity of Buc copies in Atlantic salmon contrasts with loss of the germ cell determinant in primates, rodents and axolotl
Source: BMC Evol Biol. 2016 Oct 26;16:232. doi: 10.1186/s12862-016-0809-7 (PMC5080839; doi:10.1186/s12862-016-0809-7)
Supplement: Additional file 4: Figure S3. — Partial Mexican axolotl Buc predicted from two contigs identified in the genome by Blasting against Xenopus Velo1. Sequence alignment revealed a single base deletion introducing a premature translation stop. The partial axolotl Buc shares 52 % identity with Xenopus Velo1. (DOCX 15 kb) [file 12862_2016_809_MOESM4_ESM.docx]

**Additional file 4: Figure S3**

**Partial axolotl Buc**

GTLRIRLRKLMLINCFHQYYNLTTSASPPNPHNSPQLHRSPHPHFPPHPQNSPQLHRSPHPHFPPHPQNSPQLHHSTRFRHFSSQARRTECKETQTEFRQPEPVPKDGNLGTKPKSCDAGNMTGHSSGVGTVNESISEHLESSRSSAVAAVQERDFHKNTCTSTSYRNLLPGSYAFEKER

**Contig 1**

ID C2545686, Name EPTY8IW01DR9VR
GTLRIRLRKLMLINCFHQYYNLTTSASPPNPHNSPQLHRSPHPHFPPHPQNSPQLHRSPHPHFPPHPQNSPQLHHS

**Contig 2**

ID C2444343, Name FSIRIH301EIFNF
TRFRHFSSQARRTECKETQTEFRQPEPVPKDGNLGTKPKSCDAGNMTGHSSGVGTVNESISEHLESSRSSAVAAVQERDFHKNTCTSTSYRNLLPGSYAFEKER

P G S Y A F E K E R stop

Axolotl ccaggaagctatgcatttgaaaaggag-agg**tga**ggatagagtatgggagtggtacgccc

|||||||||||||||| ||| || ||| |||| || ||||||||||| |||||| | ||

*Xenopus* ccaggaagctatgcatatgagaaagaggaggtaagaatagagtatggaagtggttcacct

P G S Y A Y E K E E V R I E Y G S G S P

Axolotl 26 ASPPNPHNSPQLHRSPHPHFPPHPQNSPQLHRSPHPHFPPHPQNSPQLHHSTRFRHFSSQ 85

+P P+ S LH P +F P PQ + ++ R PHF PHP SP +H+TRFRH+SS

*Xenopus* 55 GNPYFPYYSVALHEYPG-YFVPQPQMNTRMSR--RPHFNPHPL-SPMFYHATRFRHYSSP 110

Axolotl 86 ARRTECKETQTEFRQPEPVPKDGNLGTKPKSCDAGNMTGHSSGVG-TVNESISEHLESSR 144

RRTE KETQT+ RQ E V L T K CD GN+ HSSG+ T N+S E++E S

*Xenopus* 111 GRRTETKETQTDPRQQE-VASKKQLSTDNKGCDGGNVVYHSSGISSTGNDSNLENVEMSM 169

Axolotl 145 SSAVAAVQERDFHKNTCTSTSYRNLLPGSYAFEKE 179

S A + QERDFHKN C ST YRN+ PGSYA+EKE

*Xenopus* 170 SPATS-TQERDFHKNACNSTQYRNMPPGSYAYEKE 203
