# Supplementary material for: Multiplicity of Buc copies in Atlantic salmon contrasts with loss of the germ cell determinant in primates, rodents and axolotl
Source: BMC Evol Biol. 2016 Oct 26;16:232. doi: 10.1186/s12862-016-0809-7 (PMC5080839; doi:10.1186/s12862-016-0809-7)
Supplement: Additional file 5: Figure S4. — Conservation of the N-terminal end in A. Buc of various vertebrates, and B. BucL (−like) of zebrafish, salmon and spotted gar. Well conserved positions are shown in bold. (DOCX 15 kb) [file 12862_2016_809_MOESM5_ESM.docx]

**Additional file 5: Figure S4**

**A.**

Zebrafish Buc1 MEGINNNSQP-MGV**GQ**PH-HPV**NH**T**RPFFYVQP**PS**Q**---**PY**F--MY----**QW**P-**MN**-**PY**G

Xenopus Velo1 MNTTAPP-PEN**GQ**YS---T**N**QP**RPFFY**A**QP**TA**Q**--L**P**FPN-------P**W**Y-LGQL**Y**N

Salmon Buc1a MNNPSHS-MGV**GQ**PH-HPT**NH**T**RPFFYVQP**PT**Q**---**PY**YN-MY----**QW**N-**MN**-**PY**G

Salmon Buc2a MES**GQ**HNQRQV**NH**P**RPFFYVQP**AS**Q**---**PY**YN-MYHH--**QW**HN**MN**N**PY**N

Salmon Buc2b MES**GQ**HNQRQV**NH**P**RPFFYVQP**AS**Q**---**PY**YNNMYHHHN**QW**HN**MN**N**PY**N

Gar Buc MEEMNNPQHPSSGS**GQ**PHQHPV**NH**T**RPFFYVQP**PS**Q**---**PY**F--LY----**QW**H-**MN**N**P**FG

Coelacanth Buc MNTPPGA-AAE**G**GPY---Y**N**NS**RPFFY**A**QP**APAPPQ**P**FLN-------**QW**Y-FGHA**Y**N

**B.**

Zebrafish BucL2 43 LQLNQ**Q**FYV**P**-----------TVQ**P**F--------MP**Y**--**Q**FPTSSLCVP**Y**S

Salmon BucL1a 60 **P**Y**FY**V**QP**VQ**P**-----------**P**PP**P**F----------**Y**--**QW**-----**PMPY**N

Salmon BucL1b 58 **P**Y**FY**V**QP**--**P**-----------**P**PL**P**F----------**Y**HY**QW**-----**PMPY**N

Salmon BucL2b 62 **P**F**FY**IH**P**SQ**P**YPSQPYPSQPY**P**SQ**P**YPSQLYPSALP**Y**--**QW**-----**PMPY**N

Gar BucL 20 **P**F**FY**V**QP**SP**P**Y-----------------------FP**Y**--H**W**---HM**PMPY**H
