# Supplementary figures and images for: Multiplicity of Buc copies in Atlantic salmon contrasts with loss of the germ cell determinant in primates, rodents and axolotl
Source: BMC Evol Biol. 2016 Oct 26;16:232. doi: 10.1186/s12862-016-0809-7 (PMC5080839; doi:10.1186/s12862-016-0809-7)

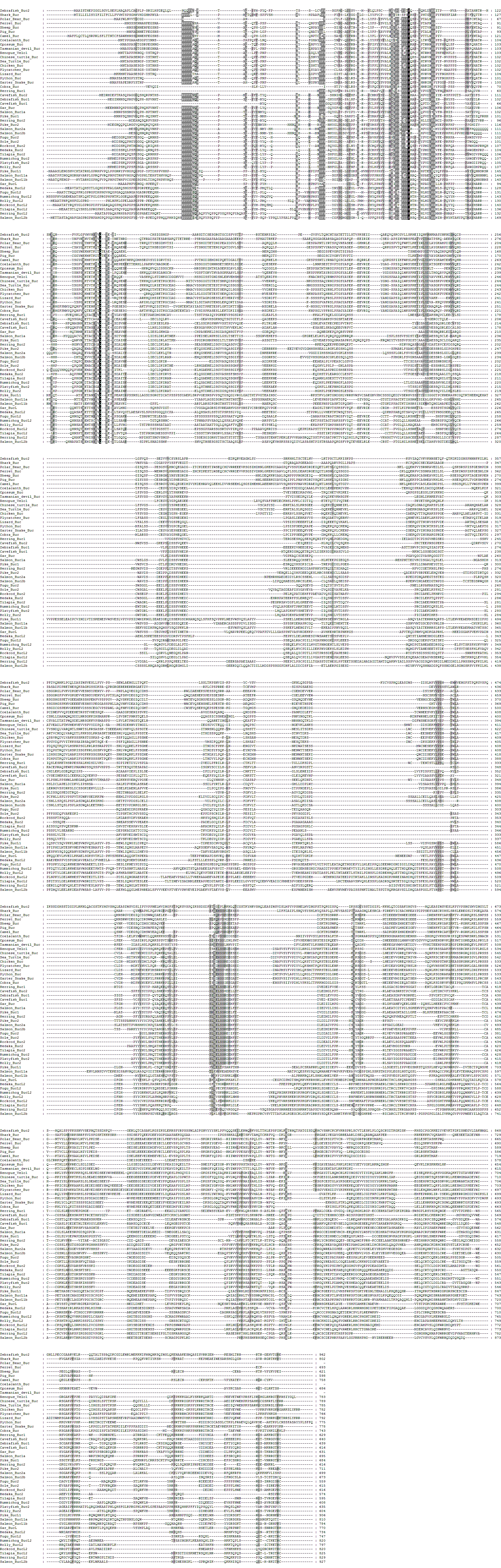

Supplement: Additional file 6: Figure S6. — Multiple Buc sequence alignments from different species were obtained using MUSLE (multiple sequence comparison by log-expectation) (www.ebi.ac.uk/Tools/msa/muscle/). The alignments were imported to GenDoc for visual inspections. (PNG 891 kb) [file 12862_2016_809_MOESM6_ESM.png]

**Additional file 8: Figure S5**


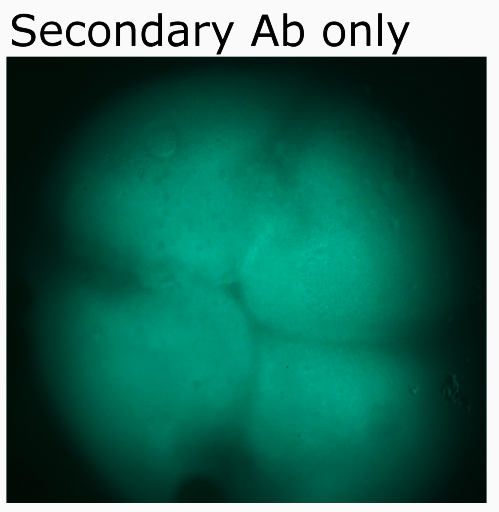

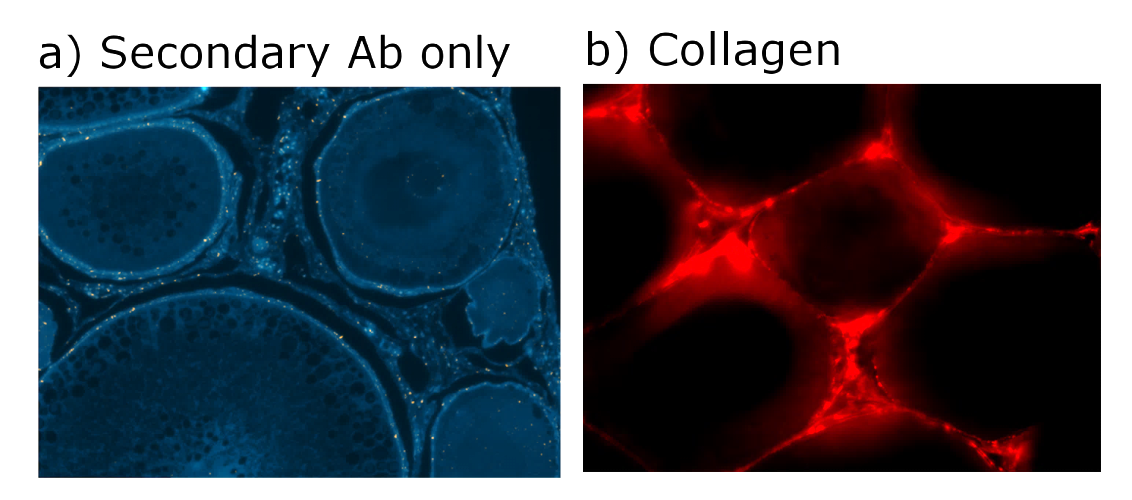

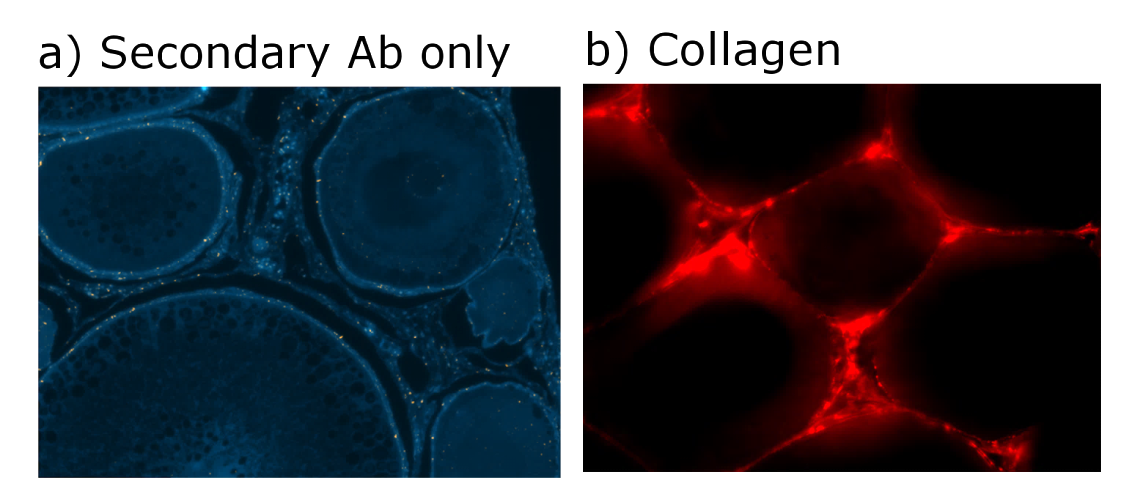


A

B

C

Supplement: Additional file 8: Figure S5. — A. Negative control (secondary antibody alone) of immunostained salmon embryo at 4-cell stage shown in Fig. 4. B. Negative control (secondary antibody alone) and C. Positive control (collagen staining) of immunohistochemical staining of ovarian sections from maturing 2-year old salmon shown in Fig. 6. (DOCX 1930 kb) [file 12862_2016_809_MOESM8_ESM.docx]
