# Supplementary material for: Multiplicity of Buc copies in Atlantic salmon contrasts with loss of the germ cell determinant in primates, rodents and axolotl
Source: BMC Evol Biol. 2016 Oct 26;16:232. doi: 10.1186/s12862-016-0809-7 (PMC5080839; doi:10.1186/s12862-016-0809-7)
Supplement: Additional file 7: Table S2. — Primer list for real-time qPCR (F-forward, R-reverse). (DOCX 16 kb) [file 12862_2016_809_MOESM7_ESM.docx]

**Additional file 7: Table S2**. Primer list for real-time qPCR (F-forward, R-reverse).

| **Gene name** | **Sequence (5’- 3’)** | **Product size** |
| --- | --- | --- |
| *buc1a* | F: TTCAACCACCAACTCAACCTT  R: GGGAACCACATAGCCAGGAT | 231 |
| *buc2a* | F: TCTGCTCTGCTGTGTGAAGA  R: AGTAGTGGTGGTGACGGACT | 94 |
| *buc2b* | F: AGCCTCTACACATCCAACAGC  R: CATCTCTGGTTCCTCCTGCT | 98 |
| *vasa* | F: CCAGTACAGAAGCATGGCATTC  R: CCGTTTTCCCAGATCCAGTCT | 82 |
| *dnd* | F: CAAGAAGGTGCTGATTGAAGC  R: GAGAGGAAGGAGGGGTTTTG | 125 |
| *eEf1-a* | F: CGCCAACATGGGCTGG  R: TCACACCATTGGCGTTACCA | 64 |
